# Supplementary material for: Oceanic adults, coastal juveniles: tracking the habitat use of whale sharks off the Pacific coast of Mexico
Source: PeerJ. 2017 May 4;5:e3271. doi: 10.7717/peerj.3271 (PMC5420197; doi:10.7717/peerj.3271)
Supplement: Figure S1 — (A) Adult female whale shark WS7 photographed on the day of tagging at Espiritu Santo Island (Table 1, Fig. 1A); (B) whale shark pup sighted at Espiritu Santo Island on 4 July 2015. Photographs copyright Carlos Aguilera Carderón (A) and Jose Maria Urbalejo Calvillo (B). [file peerj-05-3271-s001.pdf]

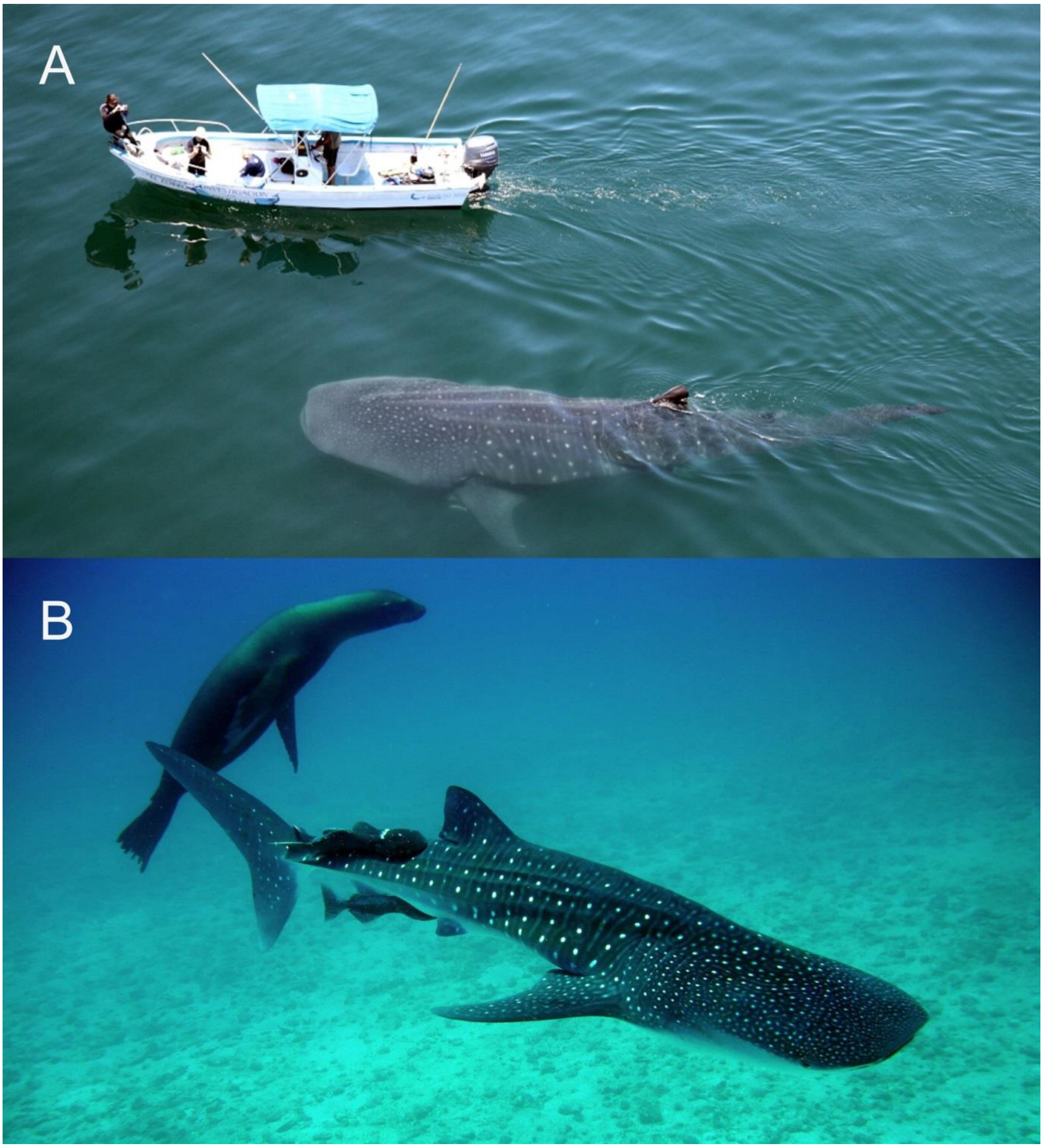

Figure S1 (A) Adult female whale shark WS7 photographed on the day of tagging at Espiritu Santo Island (Table 1, Fig. 1A); (B) whale shark pup sighted at Espiritu Santo Island on 4 July 2015. Photographs copyright Carlos Aguilera Carderón (A) and Jose Maria Urbalejo Calvillo (B).
