# Supplementary material for: Oceanic adults, coastal juveniles: tracking the habitat use of whale sharks off the Pacific coast of Mexico
Source: PeerJ. 2017 May 4;5:e3271. doi: 10.7717/peerj.3271 (PMC5420197; doi:10.7717/peerj.3271)
Supplement: Figure S2 — Along previously reported tag-specific longitudinal and latitudinal Gaussian error fields (0.16ºin longitude and 1.19ºin latitude). [file peerj-05-3271-s002.pdf]

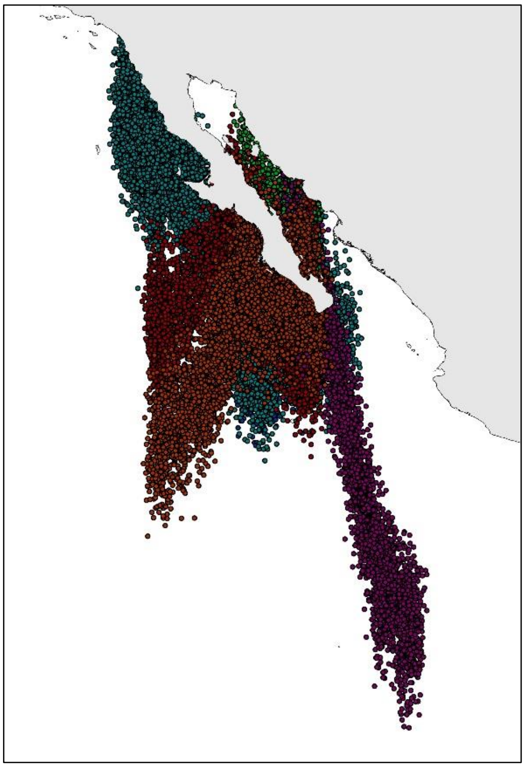

Figure S2 Resampled locations (point density data) along previously reported tag-specific longitudinal
