# Supplementary material for: Oceanic adults, coastal juveniles: tracking the habitat use of whale sharks off the Pacific coast of Mexico
Source: PeerJ. 2017 May 4;5:e3271. doi: 10.7717/peerj.3271 (PMC5420197; doi:10.7717/peerj.3271)
Supplement: Figure S3 — Averaged (0.5ºgrid) point density data (A) and environmental data (B–D); B –sea surface temperature (ºC); C –sea surface temperature gradients (ºC/10 km); D –chlorophyll a concentration. Note: these were the input data for the GLMM model. [file peerj-05-3271-s003.pdf]

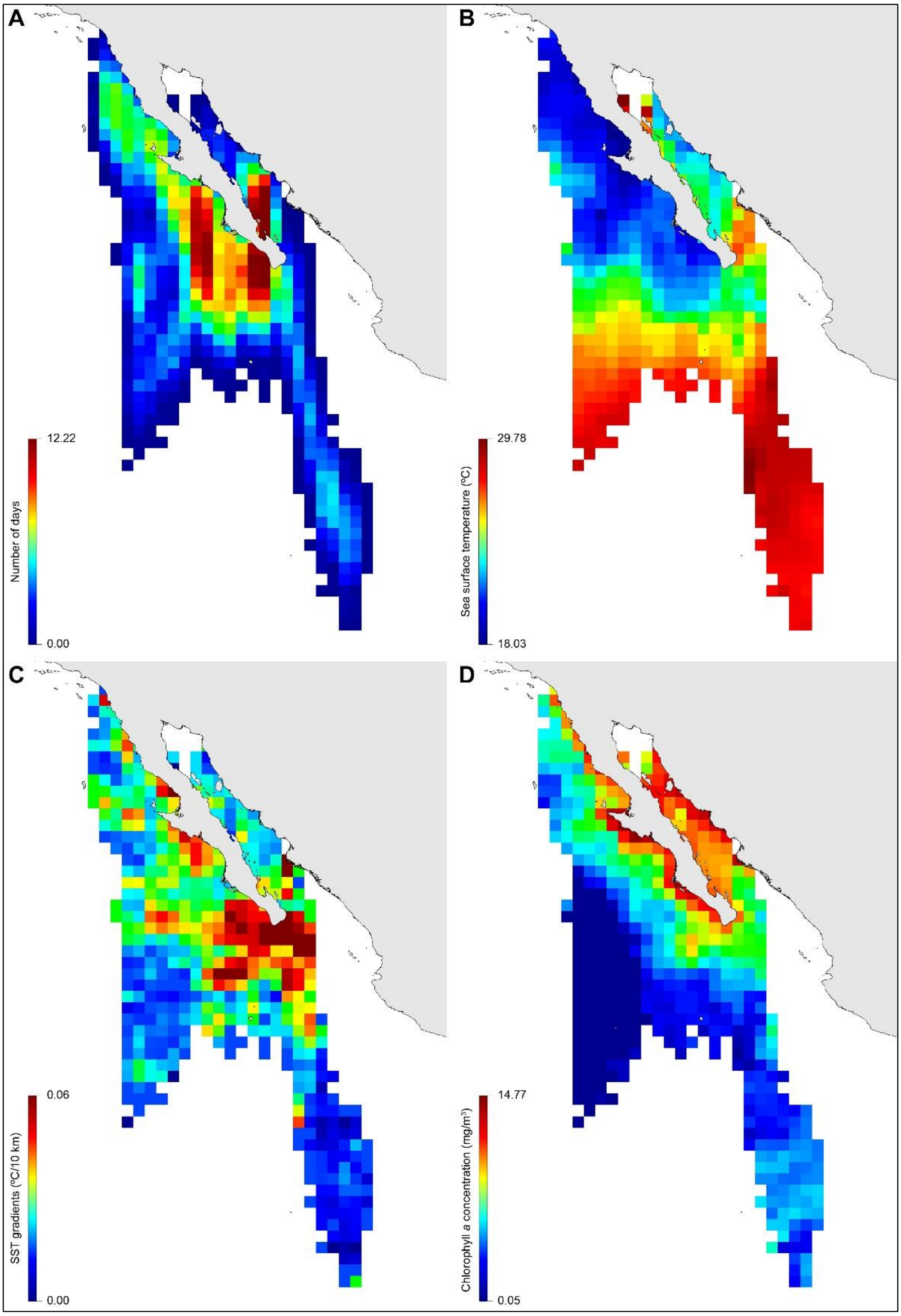

Figure S3 Averaged (0.5º grid) point density data (A) and environmental data (B-D); B – sea surface temperature (ºC); C – sea surface temperature gradients (ºC/10 km); D – chlorophyll *a* concentration. Note: these were the input data for the GLMM model.
